# Characteristics of salivary cortisol and alpha-amylase as psychobiological study outcomes in palliative care research

**DOI:** 10.1186/s12904-022-01085-1

**Published:** 2022-12-22

**Authors:** Marco Warth, Martin Stoffel, Friederike Koehler, Hubert J. Bardenheuer, Jens Kessler, Beate Ditzen

**Affiliations:** 1 Institute of Medical Psychology, Center for Psychosocial Medicine, University Hospital Heidelberg, Bergheimer Str. 20, 69115 Heidelberg, Germany; 2 Ruprecht-Karls-University Heidelberg, Bergheimer Str. 20, 69115 Heidelberg, Germany; 3 School of Therapeutic Sciences, SRH University Heidelberg, Ludwig-Guttmann-Straße 6, 66120 Heidelberg, Germany; 4 Department of Anesthesiology, University Hospital Heidelberg, Im Neuenheimer Feld 131, 69120 Heidelberg, Germany

**Keywords:** Stress response, Corticosteroid, Autonomous nervous system, Cancer, Oncology, Palliative care

## Abstract

**Background:**

Psychosocial interventions are rapidly emerging in palliative care. However, randomized trials often fail to provide evidence for their effectiveness with regard to patient-reported outcomes. Stress biomarkers could complement self-report data, but little is known about their feasibility, acceptance, and interpretability.

**Methods:**

Therefore, we designed a randomized crossover trial in which 42 patients in a palliative care unit participated in both a brief mindfulness intervention (MI) and a resting state control condition (CC) on two consecutive afternoons. On each day, we collected four saliva samples in 20-min intervals using Salivettes^©^ to determine salivary cortisol (sCort) and alpha-amylase (sAA) concentration levels. At all measurement points, self-rated well-being and stress as well as cardiovascular markers were assessed. Baseline measurements further included self-rated quality of life and clinician-rated functional status.

**Results:**

78.6% of the patients provided the maximum number of 8 saliva samples and 62.2% reported no subjective difficulties with the sampling procedures. 66.6% (sCort) and 69.6% (sAA) of all possible samples were finally included in the analysis. Xerostomia and nausea were the main reasons for missing data. Higher sCort levels were associated with higher heart rate and lower quality of life, functional status, and heart rate variability. Corticosteroid and sedative medication as well as time since last meal were identified as potential confounders. Regarding reactivity to the MI, we found an overall decrease in sCort levels over time (*b* = -.03, *p* = .01), but this effect did not differ significantly between the study conditions (*b* = .03, *p* = .21). sAA levels were higher in men than in women. Trajectories over time did not significantly differ between the two conditions (*b* = -.02, *p* = .80) and associations with other stress and health-related constructs were weak.

**Conclusions:**

Findings indicate that sCort might serve as a psychobiological outcome in future palliative care trials. However, future research should refine the exact measurement and conceptualization strategies for sCort in palliative care research. High attrition rates should be expected in patients with xerostomia or nausea.

**Trial Registration:**

Registered at the German Clinical Trials Registry (DRKS00013135) at 04/12/2017.

## Introduction

The World Health Organization defines palliative care as an approach that “improves the quality of life of patients and their families facing the problems associated with life-threatening illness, through the […] treatment of pain and other problems, physical, psychosocial and spiritual” [1]. Palliative care literature shows a vast increase in the emergence of non-pharmacological interventions to specifically address psychosocial and spiritual needs of terminally ill patients [2], but many recent studies reported difficulties in defining adequate endpoints for effectiveness. Complementing patient-reported outcomes (PROs), it could therefore be worthwhile to implement objective stress biomarkers in research on psychosocial interventions, but little is known about the feasibility, acceptance, and validity of these methods in severely ill patients.

Palliative care patients encounter a multitude of burden, including existential fears, disruptions in everyday life and cancer-related symptoms like pain and fatigue [3, 4]. Therefore, it is not surprising that research has increasingly attended to neurobiological indicators of acute and chronic stress [5]. One of the most frequently studied stress biomarkers is the hypothalamic–pituitary–adrenal (HPA) axis’ downstream hormone cortisol [6]. In presence of a stressful stimulus, the HPA axis initiates a cascade of neuroendocrine reactions until the adrenal cortex releases this glucocorticoid impacting various physiological functions. The continuing exposure to stress in severely-ill patients disturbs the regulatory feedback system of the HPA axis leading to long-term negative effects on the immune system and overall health [7, 8]. Additionally, research has recently integrated salivary α-amylase (sAA) as a non-invasive biomarker of sympathetic nervous system (SNS) activity. The enzyme was shown to be released in response to acute psychosocial or physical stressors by the salivary glands [9].

While very few studies implemented stress biomarker assessments in palliative care, more research exists on cortisol and sAA in advanced cancer patients. The majority of studies found evidence for elevated cortisol levels and flatter diurnal cortisol profiles in oncological patients [5, 8, 10–13]. Reduced variability in the diurnal regulation of cortisol was further associated with severity of fatigue, insomnia, and stressor exposure in cancer patients and survivors [14]. Moreover, initial studies found higher sAA levels in cancer patients than in healthy controls and associations with increased stress levels [15] and amount of chemotherapy [16]. However, other studies found no evidence for a dysregulation in cortisol [17] or sAA [18] in advanced cancer patients. Research including an assessment of stress biomarkers in palliative care settings is extremely rare. Only one study was identified that reported higher serum cortisol levels to predict shorter remaining life expectancy [19].

Several reviews and meta-analyses investigated the effectiveness of psychosocial interventions on cortisol in oncology and provided evidence for a reduction of cortisol levels after interventions such as cognitive-behavioral stress management (CBSM) [20], or mindfulness and religious/spiritual practices [21]. For instance, a long-term study on breast cancer patients receiving CBSM experienced significantly greater reductions in cortisol levels over 12 months compared with those in the control group [22]. Accompanying correlates of the interventions were outcomes such as increased quality of life and immune function as well as decreased fatigue, anxiety and depression. In contrast, one study [23] did not find an overall effect of a mindfulness-based intervention on cortisol as an outcome, but baseline cortisol moderated the effect of the intervention. Decreases of both cortisol and sAA were found in a study on the effects of relaxation in cancer patients [24].

In advanced cancer patients, in particular, yoga has been found to decrease cortisol levels and symptoms distress [25, 26]. Further, a study on a mindfulness-based stress reduction with advanced cancer patients [27] found short-term decreases in cortisol. Even a brief 12-min mindfulness intervention during chemotherapy showed reduced acute cortisol blunting compared to the control groups [28]. In the field of palliative care, few research has investigated the role of cortisol and sAA yet. One study reported higher random serum cortisol levels to predict shorter remaining life expectancy [19], while another study found associations of flatter diurnal cortisol slopes with symptoms like severe breathlessness [29]. Regarding interventions, merely two pilot studies were identified that included measurements of cortisol or sAA as study outcomes. Results of a pilot study with nine hospice patients [30] showed lower salivary cortisol levels after a 40-min music intervention. In contrast, Allmendinger [31] reported no differences in salivary cortisol levels compared to the control group after a single 30-min music therapy session but revealed difficulties in cortisol sampling with palliative patients, such as xerostomia and nausea.

Taken together, the measurement of cortisol and sAA in cancer patients may provide non-invasive biomarkers of disease or symptom burden independent of other pathological mechanisms and could complement PROs as endpoints in clinical trials. However, in terminal stages of a disease and particularly in palliative care, there is a paucity of systematic research, so the feasibility and interpretability of these measures remains unclear. Nonetheless, palliative care patients might have specific needs and characteristics, such as medication intake, nausea, or weakness, which need to be considered in cortisol and sAA measurement. In the present work, we therefore aimed to assess the feasibility, acceptance, and validity of salivary cortisol (sCort) and sAA assessments in palliative care in a “proof-of-concept” approach. Since past research showed beneficial effects of brief periods of interventions, we designed a randomized crossover trial investigating a 20-min mindfulness intervention with palliative care patients [28].

## Material and methods

### Study design and patients

The present study was pre-registered in the German Clinical Trials Registry (DRKS00013135) at 04/12/2017 and was approved by the local ethics committee. We conducted a randomized, crossover trial including a total of 8 measurements on two consecutive days for each participant. In addition to using patients as their own control, which was shown to be advantageous for the analysis of psychobiological data [32], crossover trials offer more statistical power, and thus, require a fewer number of participants [33]. Patients participated both in a brief pre-recorded mindfulness session (MI) at one day, and in a resting state control condition (CC) at the second. The order of the two experimental conditions was randomized across patients (computer-based block randomization), and allocation to sequence was concealed by use of sequentially numbered, opaque sealed envelopes. Blinding procedures were not feasible in this study. With regard to the analysis of reactivity, this study design represented a nested data structure, with observations at level-1 (L1), sessions at level-2 (L2), and patients at level-3 (L3). Data on the effectiveness of the MI with regards to other outcomes has been presented in a previous publication, showing that the MI led to reduction in self-rated stress and mean heart rate and to an increase in heart rate variability [34].

We recruited patients from the University Palliative Care Unit at St. Vincentius Hospital, Heidelberg, Germany. Based on an initial patient contact and the medical record, possible participants were screened for eligibility. Patients were included if they 1) received inpatient palliative treatment, 2) were assessed by the treating physician as not being in a final phase of the disease, 3) had no cognitive or hearing impairment, and 4) sufficiently spoke and understood German language.

### Procedures and intervention

**Fig. 1:**
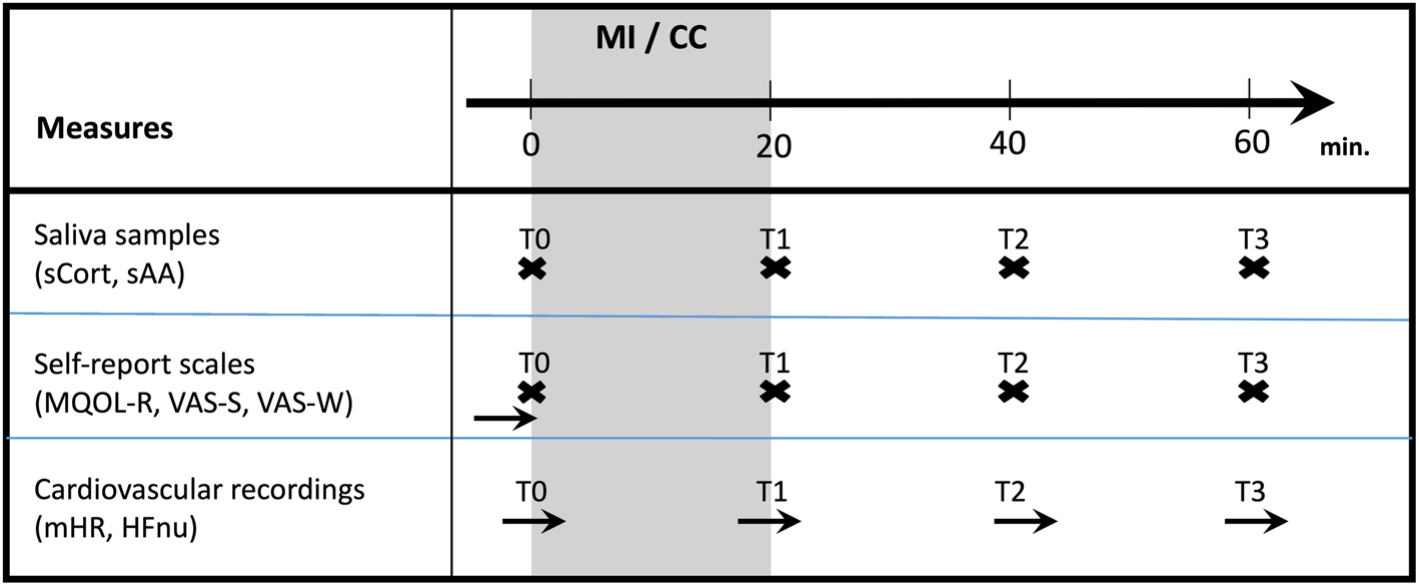
Assessments. Notes: MI = mindfulness intervention, CC = control condition, sCort = salivary cortisol, sAA = salivary alpha-amylase, MQOL-R = McGill Quality of Life Questionnaire – Revised, VAS-S = visual analogue scale “stress”, VAS-W = visual analogue scale “well-being”, mHR = mean heart rate, HFnu = high-frequency heart rate variability in normalized units

We provided information about the study goals, benefits, and potential risks, and patients had to sign the informed consent sheet if they were willing to participate. Afterwards, we opened a sealed envelope, which contained information on the treatment sequence. Appointments were made for two sessions on consecutive afternoons between 2 p.m. and 5 p.m. in order to minimize the influence of circadian variation on neuroendocrine outcomes [35]. After a questionnaire-based assessment of quality of life, a photoplethysmography (PPG) sensor was placed on the index finger of the patient’s non-dominant hand to monitor cardiovascular regulation throughout the session. During the following hour, patients were asked to provide a saliva sample and to rate their stress and well-being level every 20 min, leading to a total of four measurement points, respectively (T0-T3, Fig. 1). In the MI condition, patients were invited to listen to a 20-min recording via headphones (between T0 and T1), consisting of a breathing exercise and guided body scan meditation for supine positions, which was found to moderately improve well-being and relaxation in palliative care patients in a previous study [36]. The MI was adapted from the mindfulness-based stress reduction (MBSR) program [37] and was chosen in this study due to its brief, simple, standardized, and safe application and because cortisol was found to be sensitive to change induced by MIs in multiple settings including oncology [38]. The primary purpose of the MI was to defocus the patient's attention from symptom burden by focusing on the breath, the bodily sensations, and the present moment. Hence, it aimed at improving self-regulatory processes by increasing attentional inhibition capacities [39].

From T0-T3 in the CC as well as from T1-T3 in the MI condition, patients were asked to remain in their supine resting position. Hence, assessment procedures were identical in both sessions. Deviations from this protocol (e.g., eating, drinking, examinations, visits) were documented. Figure 1 displays the assessment plan overview. Recruitment and providing of the intervention were carried out by a research assistant who was otherwise not involved in the design and analysis of the study.

### Measures

#### Demographic and medical data

Both demographic data (e.g., age, sex, diagnoses) and information on the 24-h intake of medication on both assessment days were retrieved from the patient’s medical record. Product name and dose on the eligible day were recorded for each medication. Later, we categorized products to drug classes of analgesic, antidepressant, antipsychotic, cardiovascular, corticosteroid, hormonal, and sedative medication, and created a dichotomous variable for each class (no intake vs. intake).

#### Clinical scales

For the assessment of overall functional status, we used the physician-rated Karnofsky performance status scale (KPSS) which showed high inter-observer reliability in a validation study [40]. Patients’ performance was rated on a single 11-point scale from 0 (“dead”) to 100 (“normal, no complaints, no evidence of disease”). Moreover, we used the single item on global quality of life (gQOL) from the McGill Quality of Life Questionnaire – Revised (MQOL-R; Cohen, Sawatzky [41]). The original MQOL is a frequently used measure in patients with life-threatening diseases and its shortened and revised version showed acceptable psychometric properties [41]. Patients were additionally asked to rate their perception of acute stress and well-being on two visual analogue scales (VAS-S, VAS-W) four times per session (T0-T3) from 0–10 (with 10 indicating high stress or well-being). The use of single-item VAS was recommended in previous trials in palliative care for the brief and least burdensome assessment of psychological states [42].

#### Cardiovascular recordings

We aimed to monitor patients’ autonomic, cardiac outflow as the HPA, SAM and parasympathetic nervous system (PNS) are closely and dynamically linked to each other [43]. We used continuous PPG recordings (biosignalsplux, Lisbon, Portugal) to estimate beat-to-beat variations in heart rate in milliseconds, i.e., heart rate variability (HRV), based on a pulse wave peak detection algorithm in Kubios HRV Premium Version 3.3.0 [44]. HRV parameters were calculated for four 5-min segments parallel to the VAS and salivary assessments. We focused on mean heart rate (mHR) as a global and intuitive marker of ANS activity, and the high-frequency band in normalized units (HFnu) as a commonly reported index of vagally-mediated HRV [45] for the subsequent correlation analyses.

#### sCort and sAA assessments

We intended to collect eight saliva samples from each patient by the use of Salivette^©^ tubes (Sarstedt, Nümbrecht, Deutschland). Patients were asked to chew on the cotton wad for 1 min, which was then placed in the collection tube. If a patient refused or was unable to provide the sample (e.g., due to weakness, nausea or xerostomia), we documented the reason. After the session, all collection tubes were safely stored at -80° C at the stress biomarker lab of the Institute of Medical Psychology, University Hospital Heidelberg. Salivettes were later centrifuged according to the manufacturer’s instructions, and the extracted saliva was aliquoted and stored in polypropylene vials until performance of assays for no longer than nine months.

Cortisol was analyzed using a commercially available enzyme-linked immunosorbent assay (ELISA; DES6611; Demeditec Diagnostics, Kiel, Germany) according to the manufacturer’s protocol. sAA was analyzed using a kinetic colorimetric kit with reagents from Roche (Roche Diagnostics, Mannheim, Germany). The intra-assay coefficient of variation (CV) was 3.94% for sCort and 3.60% for sAA. The inter-assay CV was 8.90% for sAA and 7.79% for sCort.

Patients were asked to refrain from eating, drinking or other activities during sessions, if possible, and reasons for deviations from the protocol were documented. Before and after each session, we assessed possibly confounding variables in a brief interview. These variables were selected based on the recommendations provided in two methodological papers on stress biomarker assessments [35, 46]. The recommended lists of items were shortened and adapted for use in palliative care populations. The final checklist in this study consisted of items assessing either time spans in minutes (time since waking up in the morning/last nap/last meal/last drink/last toothbrush) or binary no/yes variables (caffeine intake, oral injuries, xerostomia, standing up/eating/drinking/other unintended incidents during session, subjective experience of strain due to salivary assessment). Other standard control items on nicotine/alcohol intake, symptomatic allergies or menstrual cycle were also included initially, but were excluded from the analyses due to restrictions in variance and usability in this patient population.

### Analytic strategy

#### Sample size calculations

Sample size calculations with G*Power [47] were adjusted for the change trajectories across conditions (two within-subject factors). As no effect size data for the neuroendocrine reactivity to MIs in palliative care was available, we inspected self-report and HRV data from one of our previous trials on psychosocial interventions in palliative care, and found it reasonable to assume medium-sized effects [36]. G*Power suggested *N* = 32 as the optimal sample size to detect such an effect in a four (measurements) * two (conditions) within-subjects crossover design (*f* = 0.25, α = 0.05, (1-beta) = 0.85). Accounting for a drop-out rate of approximately 30%, we aimed at recruiting *N* = 42 patients in this study.

#### Evaluation of feasibility and acceptance of sCort and sAA assessments

The evaluation of feasibility of salivary assessments was based on the patient and sampling flow data. Issues addressed in this regard were the percentages a) of patients being able to provide the maximum number of 8 samples, b) of samples not obtained due to canceled sessions or symptom distress (e.g., pain, nausea, xerostomia), c) of samples not assayed due to limited amount of liquid, and d) of samples not analyzed due to outlying values. Acceptance was defined as the percentage of patients disagreeing with the checklist item “Did you experience any difficulties with regard to the saliva sampling procedures? (no/yes)”.

#### Exploration of associations and confounders

Due to the skewness of data, all psychobiological data were log-transformed. We used a non-imputed per-protocol dataset for the exploration of associations and confounders. Outliers deviating more than three standard deviations (SD) from the sCort and sAA mean were excluded. For both sCort and sAA levels we calculated the mean within-session correlation of successive samples in the CC and the between-session correlation of baseline samples (CC and MI) as indicators of reliability. Associations with other related variables were explored either by bivariate Pearson product-moment correlations and their 95% confidence intervals (CI) in case of continuous data, or by standardized mean differences (Cohen’s *d*) and CIs in case of dichotomous predictors. Sample-level variables (L1) were the repeated measurements of VAS-S, VAS-W, mHR, HFnu, sCort, and sAA across all sessions. Session-level variables (L2) included checklist items, medication intake, and the sCort /sAA baseline scores (T0) from both sessions. Patient-level variables (L3) encompassed age, sex, KPSS, gQOL, as well as baseline levels (T0) of sCort and sAA in the CC. To provide an emphasis on the association strength of all variables, we preferred to report effect sizes and CIs rather than a multitude of hypotheses tests at this stage of the analyses. Effects that were at least medium-sized (*r* > 0.30 or *d* > 0.50, Cohen [48]) were considered relevant for further analyses.

#### Multilevel modeling of outcome data

To account for the nested data structure (observations at L1, sessions at L2, and patients at L3), multilevel modeling (MLM) was performed in the statistical environment R [49]. Primarily, data was analyzed by intention-to-treat, replacing missing values in sCort and sAA levels by means of multiple imputations. Five imputations were created with the “Amelia II” package [50] and were later pooled into a single dataset. MLM parameters were estimated with the “lme” function of the “nlme” package [51] by maximum likelihood (ML). Random intercepts were added on L2 and L3 to minimize standard errors. Treatment (TREAT) and sequence (SEQ) were dummy coded (0/1) and entered as factors. TIME was coded from 0–3 to assess linear trajectories over time. Repeatedly measured variables were averaged for each participant. Together with all remaining variables (except TIME, TREAT and SEQ), these averages were then centered on their respective grand mean and entered on L3 to obtain pure between-subject estimates [52].

Next, MLMs were built to test the role of confounders identified in the exploratory analyses. The outcomes were sCort and sAA reactivity represented by the repeated measurement of their concentrations on level 1 (L1) over the described time span, which will be referred to as “trajectories” in the following results section (contrasting sCort and sAA “levels” in the beforementioned exploratory analyses). First, full models were built which included TIME, SEQ, and the respective set of covariates. Variables were then removed in a stepwise, backwards deletion process, based on their estimate’s *p*-values. Successive models were compared with both the likelihood ratio (LR) tests for nested models and the Akaike information criterion (AIC). Given a significant LR-test, we opted to keep the model with the lower AIC. We used the models resulting from this iterative procedure and added a TIME*TREATMENT interaction as focal predictor to test the reactivity of sCort and sAA in response to the MI. Although the direction of effects was not clear to predict, we hypothesized a significantly stronger decrease in sCort and sAA in the MI compared to the CC.

Each final model was graphically assessed for violations of central model assumptions [53]. In this process, we identified skewed and leptokurtic residuals on L1 in all sCort MLMs which were caused by outlying observations from one participant. To resolve this issue, data from this participant were removed and the models affected were rebuilt. Lastly, we performed sensitivity analyses refitting the final models with the non-imputed data to assess the robustness of potentially significant findings with regard to the imputation procedure.

## Results

### Sample characteristics

*N* = 102 patients were screened between April and November 2018, and *N* = 42 (41.1%) consented to participants. On average, patients were *M* = 65.88 years old (*SD* = 13.02). The majority of patients were women (*N* = 29, 69.0%) and received treatment primarily because of an oncological disease (95%, *N* = 40). Table 1 summarizes important characteristics of patients included in this study.

**Table 1:** Sample characteristics

	***M (SD)***	***N* (%)**
**Age (*N* = 42)**	65.88 (13.01)	
**Sex (*N* = 42)**		
* Female*		29 (69.0)
* Male*		13 (31.0)
**KPSS (*N* = 42, Range: 0–100)**	40.55 (14.97)	
**gQOL (*N* = 40, Range: 0–10)**	4.75 (2.45)	
**Diagnosis (*N* = 42)**		
* Gynecological cancer*		11 (26.2)
* Pancreatic cancer*		6 (14.3)
* Gastrointestinal cancer*		5 (11.9)
* Thoracic cancer*		3 (7.1)
* Prostate cancer*		2 (4.8)
* Other cancer*		13 (31.0)
* Non-oncological disease*		2 (4.8)
**Medication (*N* = 80)**		
* Analgesic* = *YES*		72 (90.0)
* Antidepressant* = *YES*		15 (18.8)
* Antipsychotic* = *YES*		14 (17.5)
* Cardiovascular* = *YES*		53 (66.3)
* Corticosteroid* = *YES*		72 (90.0)
* Hormonal* = *YES*		14 (17.5)
* Sedative* = *YES*		13 (16.3)
**Xerostomia (*N* = 80)**		56 (71.8)
**Mouth injuries (*N* = 80)**		9 (11.5)

**Notes:** *M* mean, *SD* standard deviation, *KPSS* Karnofsky Performance Status Scale, *gQOL* global quality of life (McGill Quality of Life Questionnaire – Revised)

### Feasibility and acceptance of sCort and sAA assessments

**Fig. 2:**
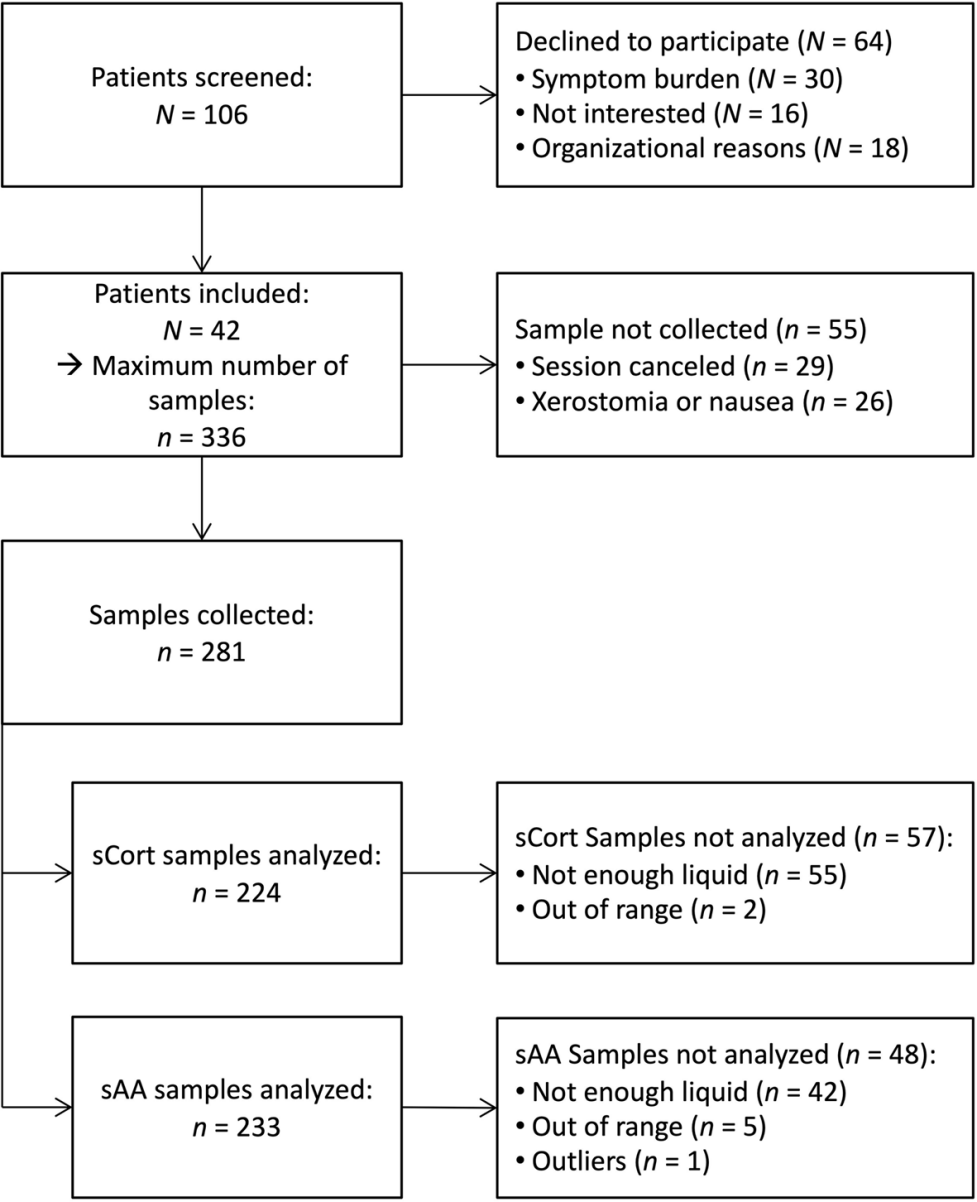
Patient flow chart. Notes: sCort = salivary cortisol, sAA = salivary alpha-amylase

The total number of patients (*N* = 42) could have provided a maximum of *n* = 336 saliva samples. In this study, it was possible to retrieve the complete number of samples from *N* = 33 (78.6%) patients. In the other *N* = 9 patients, *n* = 55 (16.3%) samples could not be collected, because sessions were either canceled (*n* = 29, 8.6%) or patients were unable to provide samples due to xerostomia or nausea (*n* = 26, 7.7%). Of the collected *n* = 281 samples, *n* = 57 (20.2%) were additionally excluded from the sCort analyses as they did not contain enough liquid for the assays (*n* = 55) or values were out of range of the assay (*n* = 2). Moreover, *n* = 48 (17.1%) samples were excluded from the sAA analyses as they did not contain enough liquid for the assays (*n* = 42), or were considered outliers (*n* = 1), or were out of range values of the assay (*n* = 5). Hence, out of the maximum number of *n* = 336, *n* = 224 (66.6%) were included in the sCort analyses, and *n* = 234 (69.6%) in the sAA analyses (Fig. 2).

Of the *N* = 37 patients who responded to the post-assessment item on acceptability, *N* = 14 (37.8%) indicated that they experienced difficulties in the saliva sampling procedures, all due to xerostomia and/or nausea. *N* = 23 (62.2%) found it acceptable to provide up to eight saliva samples over two days.

### Reliability and associations of sCort/sAA levels with related constructs

The average within-session correlation of successive sCort samples in the CC was *r* = 0.92 and the between-session correlation of baseline values was *r* = 0.78. sCort levels showed medium-sized associations with psychobiological measures including mHR (*r* = 0.46) and HFnu (*r* = -0.32). Moreover, higher sCort baseline scores were associated with lower KPSS (*r* = -0.39) and gQOL (*r* = -0.47). Correlations of sCort levels with VAS-W (*r* = -0.18) and VAS-S (*r* = 0.14) were weak but in the expected direction.

The average within-session correlation of sAA levels was *r* = 0.83, while the between-session correlation at baseline was *r* = 0.68. All associations of sAA levels with other constructs including VAS-S, VAS-W, mHR, HFNU, gQOL, and KPS were small in magnitude (all *r* < 0.30). sCort and sAA showed a small, negative correlation of *r* = -0.14 (Table. 2).

**Table 2:** Effect sizes and confidence intervals for confounders and associated variables

Variable	sCort	sAA
Within-session correlation	**r = 0,92; CI = (0,82; 0,96); N = 26**	**r = 0,83; CI = (0,66; 0,92); N = 28**
Between-session correlation	**r = 0,78; CI = (0,54; 0,90); N = 24**	**r = 0,68; CI = (0,40; 0,84); N = 26**
sAA	r = -0,14; CI = (-0,27; -0,01); N = 221	-
VAS-W	r = -0,18; CI = (-0,31; -0,05); N = 224	r = -0,05; CI = (-0,17; 0,08); N = 233
VAS-S	r = 0,14; CI = (0,26; 0,01); N = 224	r = 0,14; CI = (0,26; 0,01); N = 233
mHR	**r = 0,46; CI = (0,34; 0,57); N = 197**	r = -0,03; CI = (-0,16; 0,11); N = 204
HFnu	**r = -0,32; CI = (-0,44; -0,19); N = 200**	r = 0,10; CI = (-0,04; 0,23); N = 207
KPSS	**r = -0,39; CI = (-0,66; -0,02); N = 29**	r = -0,13; CI = (-0,47; 0,24); N = 30
gQOL	**r = -0,47; CI = (-0,71; -0,12); N = 29**	r = 0,20; CI = (-0,17; 0,52); N = 30
Age	r = 0,17; CI = (-0,21; 0,50); N = 29	r = 0,05; CI = (-0,32; 0,40); N = 30
Sex	d = 0,22; CI = (-0,49; 0,93); N = 33	**d = -0,95; CI = (-1,69; -0,22); N = 35**
Time since wake up	r = -0,13; CI = (-0,37; 0,13); N = 58	r = 0,05; CI = (-0,20; 0,30); N = 61
Time since toothbrush	**r = 0,31; CI = (0,05; 0,52); N = 57**	r = -0,07; CI = (-0,32; 0,19); N = 60
Time since last drink	r = -0,03; CI = (-0,29; 0,24); N = 54	r = 0,18; CI = (-0,09; 0,42); N = 56
Time since last meal	**r = 0,39; CI = (0,14; 0,60); N = 55**	r = -0,07; CI = (-0,32; 0,19); N = 58
Mouth injuries	**d = -0,61; CI = (-1,54; 0,31); N = 58**	d = 0,32; CI = (-0,59; 1,24); N = 61
Xerostomia	d = 0,26; CI = (-0,28; 0,81); N = 58	d = 0,02; CI = (-0,52; 0,55); N = 61
Caffeine intake	d = -0,02; CI = (-0,54; 0,50); N = 58	d = -0,13; CI = (-0,64; 0,37); N = 61
Analgesic medication	**d = 0,61; CI = (-0,32; 1,53); N = 56**	d = -0,07; CI = (-0,99; 0,84); N = 59
Antidepressant medication	d = -0,17; CI = (-0,83; 0,49); N = 56	**d = 1,26; CI = (0,56; 1,95); N = 59**
Antipsychotic medication	**d = -0,66; CI = (-1,31; -0,01); N = 56**	d = 0,22; CI = (-0,41; 0,86); N = 59
Cardiovascular medication	d = -0,01; CI = (-0,55; 0,54); N = 56	**d = -0,53; CI = (-1,07; 0,02); N = 59**
Corticosteroid medication	**d = -0,92; CI = (-1,78; -0,05); N = 56**	d = -0,15; CI = (-1,00; 0,69); N = 59
Hormonal medication	d = 0,26; CI = (-0,46; 0,97); N = 56	**d = -1,42; CI = (-2,15; -0,69); N = 59**
Sedative medication	**d = 0,75; CI = (-0,11; 1,61); N = 56**	**d = -0,75; CI = (-1,51; 0,01); N = 59**

**Notes:** *sCor*t salivary cortisol, *sAA* salivary alpha amylase, *VAS-W* visual analogue scale – wellbeing, *VAS-S* visual analogue scale – stress, *mHR* mean heart rate, *HFnu* High frequency heart rate variability in normalized units, *KPSS* Karnofsky Performance Status Scale, *gQOL* global quality of life (McGill Quality of Life Questionnaire – Revised)

### Confounding variables

Cortisol baseline levels showed a weak correlation with age (*r* = 0.17) and differences between sexes were small (*d* = 0.22). Exploration of potential confounders assessed via checklists revealed sCort baseline levels to be associated with time since teeth brushing (*r* = 0.31), time since last meal (*r* = 0.39), and the reporting of mouth injuries (*d* = -0.61). Moreover, sCort baseline levels were lower if patients were on corticosteroid (*d* = -0.92) or antipsychotic medication (*d* = -0.66), and higher if patients took analgesic (*d* = 0.61) or sedative medication (*d* = 0.75, Table 2). Analyzing these confounders simultaneously and hierarchically revealed that sedative (*b* = 0.69, *p* = 0.04) and corticosteroid medication (*b* = -0.92, *p* = 0.03), as well as time since last meal (*b* < 0.01, *p* < 0.01) remained important covariates for multilevel modeling of sCort trajectories over time (Table 3, M1). Other predictors were deleted in the stepwise model building process.

sAA baseline levels were unrelated to age (*r* = 0.05), but scores were higher in men than in women, with a large between-groups effect size of *d* = -0.95. In addition, sAA baseline levels depended on the intake of antidepressant (*d* = 1.26), cardiovascular (*d* = -0.53), hormonal (*d* = -1.42), and sedative medication (*d* = -0.75) in explorative, bivariate analysis. Effect sizes of all other potential confounders assessed via checklist items were small (Table 2). In the course of multilevel modeling, all potential confounders concerning medication were deleted and sex remained the only covariate relevant for sAA trajectories over time (*b* = -0.83, *p* = 0.01; Table 3, M3).

### sCort and sAA reactivity to mindfulness intervention

**Fig. 3:**
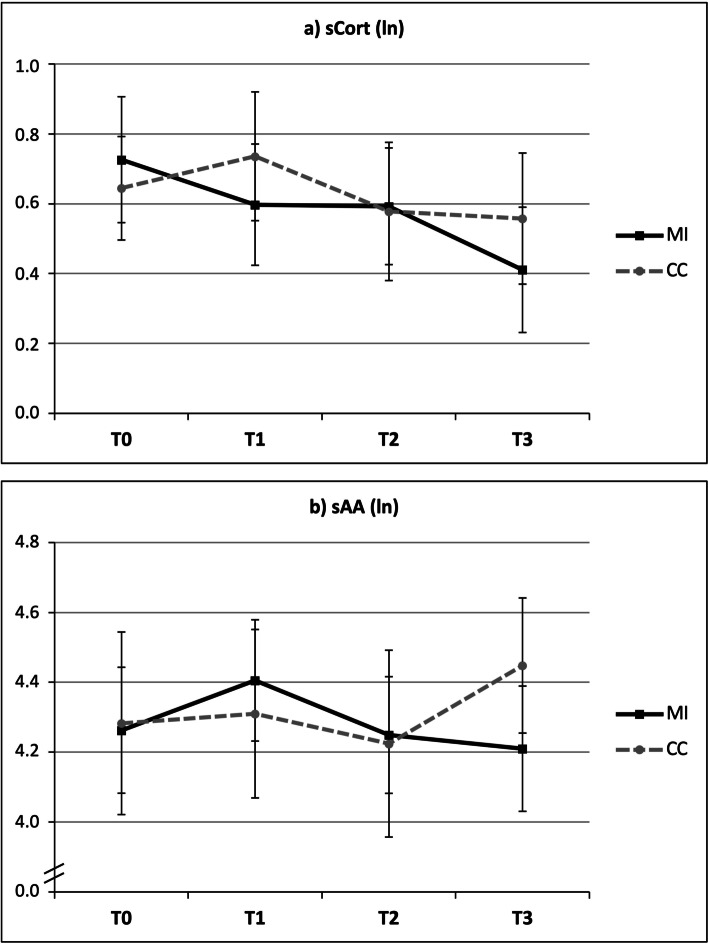
Means and standard errors of sCort and sAA trajectories over time by treatment. Notes: sCort = salivary cortisol, sAA = salivary alpha-amylase, MI = mindfulness intervention, CC = control condition, ln = log-transformed

These final sets of predictors were used as covariates for the subsequent testing of differences between the MI and CC in the sCort and sAA trajectories over time (T0-T3), which would serve as indicators of sCort or sAA reactivity in response to the MI. Preliminary testing of potential carryover effects was statistically not significant for sCort (*T* = -0.94, *p* = 0.35) and sAA (*T* = -1.44, *p* = 0.16). Means and standard errors of sCort (a) and sAA (b) trajectories over time are displayed in Fig. 3 (non-imputed, observed data). Table 3 (M2 and M4) depicts the results of multilevel modeling of reactivity analyses showing that the TIME*TREATMENT interaction was statistically not significant for both sCort (*b* = 0.03, *p* = 0.21, M2) and sAA trajectories (*b* = -0.02, *p* = 0.80, M4). Sensitivity analyses with the non-imputed dataset confirmed this pattern of non-significant TIME*TREATMENT interactions for sCort (*b* = 0.01, *p* = 0.56) and sAA trajectories (*b* = -0.08, *p* = 0.26). However, sCort levels significantly decreased over time regardless of the provided intervention, which was reflected by a significant main effect of time in both the imputed (*b* = -0.05, *p* < 0.01) and non-imputed (*b* = -0.07, *p* < 0.01) data in M2. This finding was further confirmed by the statistically significant decrease over TIME in the M1 model without the interaction (*b* = -0.03, *p* = 0.01).

**Table 3:** Multilevel modeling of psychobiological data

**Fixed effects**	**M1: sCort (covariates)**		**M2: sCort (+ treatment)**		**M3: sAA (covariates)**		**M4: sAA (+ treatment)**	
	**Est**	***p***	**Est**	***p***	**Est**	***p***	**Est**	***p***
INTERCEPT	**0.821**	**0.000**	**0.974**	**0.000**	**4.679**	**0.000**	**4.745**	**0.000**
SEQUENCE (0 = MI—> CC, 1 = CC—> MI)	-0.106	0.639	-0.145	0.506	-0.124	0.669	-0.206	0.459
TIME (linear: 0, 1, 2, 3)	**-0.033**	**0.013**	**-0.048**	**0.009**	0.010	0.732	0.028	0.484
TREAT (0 = CC, 1 = MI)	-	-	-0.048	0.678	-	-	-0.037	0.799
SEX (0 = male, 1 = female)	-	-	-	-	**-0.826**	**0.010**	**-0.761**	**0.015**
MED.SEDA (0 = no, 1 = yes)	**0.691**	**0.038**	**0.682**	**0.035**	-	-	-	-
MED.CORT (0 = no, 1 = yes)	**-0.920**	**0.030**	**-0.820**	**0.035**	-	-	-	-
TIME SINCE LAST MEAL (minutes)	**0.001**	**0.002**	**0.001**	**0.001**	-	-	-	-
TIME*TREAT	-	-	0.032	0.214	-	-	-0.015	0.798
**Random effects (variances)**								
INTERCEPT L3 (across patients)	0.303	-	0.271	-	0.631	-	0.613	-
INTERCEPT L2 (across sessions)	0.186	-	0.198	-	0.189	-	0.200	-
Residual variance	0.057	-	0.059	-	0.331	-	0.334	-
**Model fit**								
AIC	244.104	-	271.232	-	728.881	-	771.726	-
BIC	276.688	-	311.973	-	755.215	-	806.027	-
***N***								
Observations (L1)	276	-	300	-	318	-	334	-
Sessions (L2)	69	-	75	-	80	-	84	-
Patients (L3)	38	-	38	-	40	-	42	-

**Notes:** *sCort* salivary cortisol (log-transformed), *sAA* salivary alpha-amylase (log-transformed), *M* model, *L* level, *Est* Estimate, *MI* mindfulness intervention, *CC* control condition, *MED.SEDA* sedative medication, *MED.CORT* corticosteroid medication, *AIC* Akaike Information Criterion, *BIC* Bayesian information criterion, bold effects were statistically significant on the level of *p* < 0.05

## Discussion

In a previous publication from this study, we found a decrease in self-rated stress and heart rate as well as an increase in heart rate variability in response to the MI [34]. While these study outcomes have been successfully implemented in other palliative care studies before, we also implemented the collection and analysis of saliva-based stress biomarkers that have not been examined in this context before. Hence, to our knowledge this was the first study to systematically address the issues of feasibility, acceptance, validity, and change sensitivity of salivary stress biomarker assessments in palliative care. Our findings suggest that – despite previously reported challenges [31] – repeated salivary biomarker assessments via Salivettes were generally feasible in severely ill patients, as almost 4 in 5 (78.6%) patients were able to provide the maximum number of eight samples on two consecutive days. In line with previous research, however, the majority of patients reported symptoms of xerostomia or nausea [54], which were major reasons for a substantial discrepancy between the number of samples collected and samples successfully assayed, as either the cotton swab could not be tolerated long enough or the salivary glands did not produce enough liquid. Hence, the final number of samples analyzed should rather be expected to be two thirds of the maximum possible number for both sCort (66.6%) and sAA (69.6%). Also, more than one third of patients (37.8%) reported subjective difficulties in the collection of saliva samples, again mostly due to xerostomia and nausea. Hence, if researchers aim at increasing the acceptance of their salivary biomarker assessments in future research, they may consider excluding patients with dryness of mouth or nausea after an initial screening.

In addition to the issues of feasibility and acceptance, we analyzed associations of stress biomarkers with presumably related constructs. Regarding sCort baseline levels, we observed medium-sized, negative associations with functional status (KPSS, *r* = -0.39) and global quality of life (gQOL,* r* = -0.47). Hence, a higher baseline cortisol score corresponded to a negative global evaluation of health status, both in the subjective (gQOL) and clinician-rated (KPSS) domain. Taking into account that these correlations were merely based on a single measure, salivary cortisol assessments may be considered to complement standard diagnostic procedures in palliative care settings. Moreover, cortisol showed the expected associations with other physiological markers. Patients with higher sCort levels also had a higher mean heart rate (mHR, *r* = 0.46) and lower vagally-mediated HRV (HFnu, *r* = -0.32), both of which were found to predict mortality and negative health outcomes in severely ill patients [55, 56]. Correlations of sCort levels with self-reported stress and well-being were weak but still in the expected direction. Taken together, the data provides first evidence for the reliable and valid use of sCort level as a marker of HPA-activity in palliative care. However, it needs to be considered that these findings are preliminary as they were based on multiple bivariate correlations only.

The stepwise exploration of potential confounders revealed time since last meal and the intake of both sedative and corticosteroid medication as important covariates of sCort. Particularly, corticosteroids (e.g., dexamethasone) are frequently prescribed in inpatient palliative care (90.0% in our sample) and our data showed the intake to be associated with a lower concentration of endogenous cortisol levels in saliva. Methodologically, an unbalanced and high proportion of patients on corticosteroids medication may limit the potential to statistically control for this confounder. Therefore, future researchers may carefully evaluate whether it could make sense to add an inclusion criterion with regard to corticosteroid intake (all patients or no patients), and thus to eliminate confounding due to this medication class.

Cortisol assessments have been proposed as potential markers for improvement in trials testing MIs, but previous studies mostly implemented multiple-session programs such as MBSR in disciplines other than palliative care [38] and used heterogeneous strategies for collection and analysis of biomarker data [57]. Possibly due to the very brief and single-session intervention in the present study, we were unable to show a distinct reactivity of sCort in response to the MI, and thus, to replicate these findings. However, cortisol trajectories significantly decreased over time regardless of the intervention provided, which may either be explained by the general decrease in diurnal cortisol [35] or by an initially arousing effect of the assessment procedures which patients later adjusted to. Hence, while we found a moderate decrease in self-rated stress in response to the MI in a previous analysis [34], the psychobiological changes observed in the sCort reactivity may rather be driven by diurnal variation or by the experimental setup itself than by the very brief MI.

While preliminary conceptual evidence favoring the use of sCort as a stress biomarker in palliative care was found in this study, results on the validity and interpretability of sAA were less straightforward. Effect sizes on associations between sAA levels and presumably related constructs were weak, including self-rated well-being, stress, and quality of life, as well as cardiovascular outflow and clinician-rated functional status. Some previously described methodological pitfalls associated with salivary flow rate or the use of cotton swabs in sAA assessments [58] may also apply to the present study. In palliative care patients, however, methodological challenges are likely to occur across various assessment and collection procedures. For instance, the ‘passive drool method’ [59] as one potential alternative would have required an even higher amount of spontaneous and unstimulated saliva production. Moreover, it is possible that area under the curve (AUC, Pruessner, Kirschbaum [60]) indices could provide better convergent validity for sAA measures than the analysis of single (baseline) values. While these indices increase the difficulties in dealing with large proportions of missing data, ad-hoc analysis of our data revealed medium-sized associations between the sAA AUCg and both HFnu (positive) and KPSS (negative). Hence, it could be worthwhile to implement methods that allow for unbiased AUC analysis in future studies interested in the prognostic properties of sAA assessments.

A number of potential confounders were identified for sAA levels in the first explorative analysis step including antidepressant, cardiovascular, hormonal, and sedative medication intake. When analyzing all covariates simultaneously and hierarchically, however, only sex remained as a significant covariate for sAA trajectories. The pattern of sex differences in sAA levels confirmed previous findings with men having higher sAA concentrations than women [61]. The individual sAA trajectories over time did not systematically differ between the two study conditions which may be due to large within- and between-subjects variance in this biomarker. Therefore, our data does not provide support for the use of sAA reactivity as an indicator of acute SAM-system change in response to MIs.

This study presents unique and novel insights into the potential of stress biomarker assessments in palliative care, but also faced several limitations. First and as expected, we observed a high proportion of missing data due to canceled sessions, xerostomia, nausea, or issues occurring during the performance of assays. It should be noted that acquiring data on patient and sample flow was part of the present research question with regard to feasibility and the presented findings may help to guide future studies in the conceptualization of appropriate study designs. However, the low number of actually available samples reduces the generalizability of the subsequent findings. In particular, the assessment and analysis of sAA in the present study suffered from a very limited amount previous research. Although one study found high correlations in sAA concentrations between the Salivette and passive drool method in healthy adults, little is known about the optimal collection and handling procedures in clinical samples. Hence, more research e.g. in general cancer populations is needed, before the implementation of sAA measurements in palliative care research can be recommended. Second, the use of crossover designs involves certain methodological characteristics that need to be considered. Therefore, we implemented a washout period of one day which we believed to be sufficient for the short-term effects of the very brief MI. We also tested for carryover effects and controlled for sequence effects in all multilevel models examining the effectiveness of the MI. Moreover, study’s findings concerning feasibility and acceptance are limited by the fact that we could not find any previous literature that helped us guiding an a priori and straightforward definition. In the present study, both feasibility and acceptance specifically referred to the assessments and collection procedures of sCort and sAA, and not to the implementation of the MI, for which more standard literature would have been available. Hence, the presented criteria were developed rather inductively and could have been chosen differently. Finally, the associative analyses in this study had an explorative nature. Due to a paucity of previous research on sCort and sAA assessments in palliative care, we aimed at exploring associations and confounders to facilitate future research in the field.

## Conclusions

This study aimed at investigating the measurement characteristics of sCort and sAA levels and MI reactivity as biomarkers of stress in palliative care research. Our findings indicate that repeated salivary assessments were generally feasible and accepted in the majority of terminally-ill patients. However, the sample flow data made clear that a high collection rate does not necessarily entail a complete and unbiased dataset. Symptoms of xerostomia and nausea not only led to the cancelation of assessments or complete sessions, but samples collected from patients with severe dryness of mouth, for instance, often contained too little liquid for biomarker assays. Imputation methods can help to deal with such a dataset which in this study was based on approximately two thirds of the maximum possible number of samples. Our data did not provide strong evidence for a valid use of sAA levels or reactivity as a stress biomarker in palliative care. However, based on the observed associations of sCort levels with quality of life, functional status, and cardiovascular markers, sCort levels may serve well as a secondary outcome in future trials on psychosocial interventions or to complement standard diagnostic procedures on palliative care units. Future researchers are encouraged to closely monitor the identified confounding variables to further study the potential of stress biomarkers in palliative care, and to test alternative measuring strategies and conceptualizations of sCort, such as the repeated measurement of the cortisol awakening response or diurnal slope, evaluating their usefulness in clinical trials.

## Data Availability

The datasets used and/or analyzed during the current study are available from the corresponding author on reasonable request.
